# Subacute reconstruction using flap transfer for complex defects of the upper extremity

**DOI:** 10.1186/s13018-020-01647-0

**Published:** 2020-04-07

**Authors:** Yongqiang Kang, Xiaoyun Pan, Yongwei Wu, Yunhong Ma, Jun Liu, Yongjun Rui

**Affiliations:** 1 Department of Traumatic Orthopedics, Wuxi Ninth People’s Hospital affiliated to Soochow University, NO.999 Liangxi Road, Wuxi City, 214062 Jiangsu Province China; 2 Orthopaedic Institute, Wuxi Ninth People’s Hospital affiliated to Soochow University, Wuxi, Jiangsu China

**Keywords:** Upper extremity, Flap transfer, Reconstruction, Open fracture

## Abstract

**Background:**

Despite advances in microsurgical techniques of flap transfer, complex upper extremity trauma reconstruction remains a challenge for surgeons. This study aimed to present the outcomes in using flaps in the subacute reconstruction of complex upper extremity injuries.

**Methods:**

From July 2013 to December 2016, 35 patients ranging in age from 23 to 69 years with complicated upper extremity traumatic injuries were treated using flap reconstruction in subacute period. The number and causes of injury were 12 machine crush injuries, 18 machine strangulation injuries, two chainsaw accidents, two traffic accidents, and one incident of heavy bruising. Thirty-five patients underwent flap procedures, including 24 anterolateral thigh flaps (68.57%), five latissimus dorsi flaps (14.29%), and six lateral arm flaps (17.14%). Flap sizes ranged from 3 × 4 to 42 × 16 cm^2^. The mean time of flap reconstruction was 14 days (range 5–29). During postoperative follow-up, flap appearance, sensory recovery, scarring and satisfaction were assessed.

**Results:**

The overall flap survival rate was 94.3%.Two flaps developed partial necrosis, both of which were later treated with skin grafting. Traumatic wound infections occurred in three patients. All upper limb injuries were completely covered. The follow-up periods ranged from 18 to 62 months with an average of 2.9 months. All skin flap textures were soft with varying degrees of pigmentation. Flap sensory recovery was S_1_ in three cases, S_2_ in eight cases, S_3_ in 15 cases, and S_4_ in nine cases. There were no donor site complications other than three cases with scar hyperplasia.

**Conclusions:**

The severe upper limb soft tissue defects still achieved satisfactory function and appearance with negligible complications and low amputation rates during the subacute period.

## Introduction

Complex traumatic upper extremity injuries are frequently characterized by compromised local vasculature. Some extensive defects are difficult or even impossible to repair successfully. Flap transfer for upper limb reconstruction requires more than just wound coverage, unlike lower limbs. The goals of upper extremity reconstruction are optimization of functional and esthetic outcomes while minimizing donor damage as well as prevention of infections while providing adequate soft tissue protection of vital structures. In these instances, free tissue transfer is required to provide adequate soft tissue coverage. Many types of skin flaps can be used as an option for wound coverage [1, 2].

Howard et al. reviewed survival rates of anterolateral thigh flaps for upper extremity trauma reconstruction and found encouraging rates ranging from 90 to 100% [3]. Nevertheless, the timing of tissue transfer has been hotly debated for years. It was originally suggested by Godina that microsurgical reconstructions of wounds within 3 days would decrease the rate of infection and shorten hospital stay and bone-healing time [4]. Since then, 72 h has been considered the “golden window” for flap reconstruction [5]. However, such patients have relatively serious injuries and have suffered a large amount of blood loss. The first stage of tissue transplantation not only greatly lengthens the operation time, but also is a physical stressor because the patients’ general conditions are not always suitable for emergency surgery. Moreover, emergency doctors must be skilled in microsurgical techniques. With improvements in preoperative management, especially with the application of vacuum sealing drainage (VSD) negative pressure aspiration, the time of flap reconstruction beyond the golden window still achieved satisfactory outcomes [6]. Derderian et al. reported a 25-year experience for microvascular free tissue transfer for traumatic defects of the upper extremity [7]. The authors suggested that the preferred timing for microvascular free tissue transfers to the upper extremity was 6 to 21 days post injury because of the decreasing rate of flap failures, re-explorations, infections, osteomyelitis, and nonunions. In a word, the timing of tissue transfer remains a controversial topic. In this report, we presented our experience of using flap transfer for traumatic defects for the upper extremity in the subacute period, aimed to provide more clinical evidence for the good outcomes of subacute reconstruction.

## Patients and methods

We performed a retrospective study of patients who came to our hospital for treatment of complex upper limb trauma with flap transfers between July 2013 and December 2016. Only cases reconstructed during the subacute phase were included. There were 25 male and 10 female patients, whose ages ranged from 23 to 69 years old. Thirty-five flaps were transferred in 35 upper extremities for delayed reconstruction in the subacute period, with an average time to reconstruction of 14 days (range, 5–29 days). All patients sustained complex upper extremity injuries. The major etiologies were machine crush injuries (30 patients).

Initial debridement of devitalized and contaminated tissues was performed under loupe magnification and tourniquet control. Debridement was performed by a senior doctor followed by irrigation with 9 L of solution. After complete debridement, the fractures were fixed internally in one stage, and the remaining wounds were covered with VSD. Flap transplantation occurred in the subacute period. We selected the corresponding flap based on the wound size and muscle soft tissue defect. The upper extremity reconstructive tissue transfer used was 24 anterolateral thigh flaps (68.57%), five latissimus dorsi flaps (14.29%), and six lateral arm flaps (17.14%). Five defects of the major arteries were reconstructed using ALT flaps with a flow-through pattern. Four defects were reconstructed using the ALT flaps with split skin paddles flaps. Three defects were reconstructed using ALT flaps without the fascia lata. Preoperative CTA and CDS were used to locate the bilateral thigh perforator of patients with anterolateral femoral skin flap transplantation. Data collected included the type of tissue transfer, postoperative flap complications, infection data, and outcomes (Tables 1, 2, and 3).

**Table 1:** Patient anterolateral thigh (ALT) flap reconstruction

Sex	Age (years)	Cause of injury	Associated fracture	Associated injury	Time to debridement (hours)	Fracture fixation	Number of washouts	Time to flap (days)	Type flap	Flap failure/comments	Infection
Male	45	Machine strangulated	Radius, ulna	Radial artery, ulnar artery	7	Internal	1	6	Radial flow-through		None
Male	27	Machine strangulated	Carpals	Radial artery, ulnar artery	6	Internal, external	1	8	Radial flow-through		None
Male	24	Machine strangulated	Radius, ulna, metacarpal		3	Internal	2	21	Single		None
Male	54	Machine crushed	Ulna		2	Internal	1	18	Single		None
Male	32	Machine crushed	Radius, ulna, carpals	Radial artery, radial nerve	4	Internal, external	4	13	Single		None
Male	57	Machine strangulated	Radius, ulna		10	External	4	5	Split skin paddles flap	Partial flap loss	Superficial infection
Female	43	Machine crushed	Radius, ulna	Radial artery	7	Internal	3	29	Single	Microvascular re-exploration	None
Female	48	Machine strangulated	Radius		4	Internal	3	22	Radial flow-through		None
Male	23	Machine strangulated	Carpals, radius	Radial artery	2	Internal, external	1	7	Single		None
Male	52	Hot machine crushed	Radius, ulna	Radial artery, radial nerve	5	Internal	4	26	Single	Microvascular re-exploration	Superficial infection
Male	54	Machine strangulated	Radius, ulna, carpals		4	Internal	1	9	Single, without fascia lata		None
Male	26	Machine strangulated	Humerus, radius, ulna		6	Internal	1	27	Split skin paddles flap		None
Male	42	Machine crushed	Ulna		4	Internal	1	15	Single		None
Male	45	Machine strangulated	Humerus	Brachial plexus, brachial artery, radial artery	3	Internal	0	8	Radial flow-through		None
Female	45	Machine strangulated	Radius, ulna		8	Internal	0	7	Single		None
Male	65	Machine strangulated	Humerus, radius, ulna	Radial nerve	6	Internal	1	24	Single		None
Male	50	Machine strangulated	Radius, ulna		9	Internal	1	17	Single		None
Female	65	Machine crushed	Radius, ulna	Ulnar artery	2	Internal, external	1	11	Single, without fascia lata		None
Male	69	Heavy bruised	Humerus	Brachial artery, radial artery, ulnar artery	6	Internal	0	10	Radial flow-through		None
Female	62	Machine crushed	Humerus, radius, ulna	Radial nerve	5	Internal	1	12	Single, without fascia lata		None
Male	42	Machine strangulated	Radius, ulna, carpals	Radial artery	3	Internal, external	0	8	Split skin paddles flap		None
Female	46	Machine crushed	Radius	Radial artery	8	Internal	0	6	Split skin paddles flap		None
Male	27	Machine strangulated	Radius, ulna		2	Internal	1	12	Single		None
Male	23	Machine strangulated	Radius, ulna		6	Internal, external	0	5	Single		None

**Table 2:** Patient latissimus dorsi flap reconstruction

Sex	Age (year)	Cause of injury	Associated fracture	Associated injury	Time to debridement (hours)	Fracture fixation	Number of washouts	Time to flap (days)	Type flap	Flap failure/comments	Infection
Female	52	Hot machine crushed	Ulna	Ulnar artery, ulnar nerve	2	Internal	3	21	Free flap	Microvascular re-exploration	None
Male	59	Hot machine crushed	Humerus, ulna		6	Internal	3	28	Island flaps		None
Male	42	Machine strangulated	Humerus, ulna, radius		2	Internal, external	1	16	Island flaps	Partial flap loss	None
Male	37	Machine strangulated	Radius, ulna	Radial artery, ulnar artery	5	Internal	1	6	Island flaps		None
Female	41	Traffic accidents	Humerus, ulna, radius	Brachial artery	8	Internal, external	3	27	Island flaps		Superficial infection

**Table 3:** Patient lateral arm flap reconstruction

Sex	Age (year)	Cause of injury	Associated fracture	Associated injury	Time to debridement (hours)	Fracture fixation	Number of washouts	Time to flap (days)	Type flap	Flap failure/comments	Infection
Female	54	Machine crushed	Ulna	Ulnar artery, ulnar nerve	2	Internal	3	21	Free flap		None
Male	59	Chainsaw	Radius	Radial artery	6	Internal	1	8	Island flaps		None
Male	40	Chainsaw	Ulna, carpals	Ulnar artery, ulnar nerve	2	Internal, external	1	6	Free flap		None
Male	38	Machine crushed	Radius, ulna	Radial artery, ulnar artery	5	Internal	1	14	Island flaps		None
Male	47	Traffic accidents	Metacarpal, carpals		4	Internal	2	10	Free flap		None
Female	52	Machine strangulated	Radius	Radial artery	3	Internal	1	12	Free flap		None

## Results

The defect sizes were from 3.5 × 5.0 cm to 15 × 40 cm. The dimensions of the ALT flaps ranged from 8 to 42 cm in length and 6 to 16 cm in width. Latissimus dorsi flaps ranged from 28 to 36 cm in length and 7 to 9 cm in width. The lateral arm flaps ranged from 4 to 10 cm in length and 3 to 6 cm. Parts of the defects were grafted with split-thickness skin secondarily. There were anastomoses of vessels in 24 cases with ALT flaps. There were 18 cases of anastomosis of the radial artery and accompanying vein, and six cases of the anastomosis of ulnar artery and the accompanying vein.

All but two of 35 flaps survived completely with three flaps requiring microvascular re-exploration. The hospital stays ranged from 12 to 103 days (average, 39 days). The two lost partial flaps were an anterolateral thigh flap and a latissimus dorsi flap, both because of deficient blood supply. Traumatized wound infections occurred in three patients, and further debridement was performed in two patients except one healed after five change dressings at the bedside. Two resulted from progressive muscle and soft tissue necrosis of the wounds. Rehabilitation training was performed by physical therapists within 7 days of admission. The follow-up periods ranged from 18 to 62 months. There were no other donor site complications except three cases with scar hyperplasia. One patient had chronic infection with a draining sinus after 13 months. Washout was performed after rehospitalization, and the sinus healed. No bacterial growth was observed in secretions. Three patients, all with latissimus dorsi flaps, had S_1_ sensation; eight patients had S_2_ sensation, including two latissimus dorsi flaps, four ALT flaps, and two lateral arm flaps; 15 patients, two with lateral arm flaps and 13 with ALT flaps, had S_3_ sensation; and nine patients had S_4_ sensation, including two lateral arm flaps and seven ALT flaps.

## Representative cases

### Case 1

**Fig. 1:**
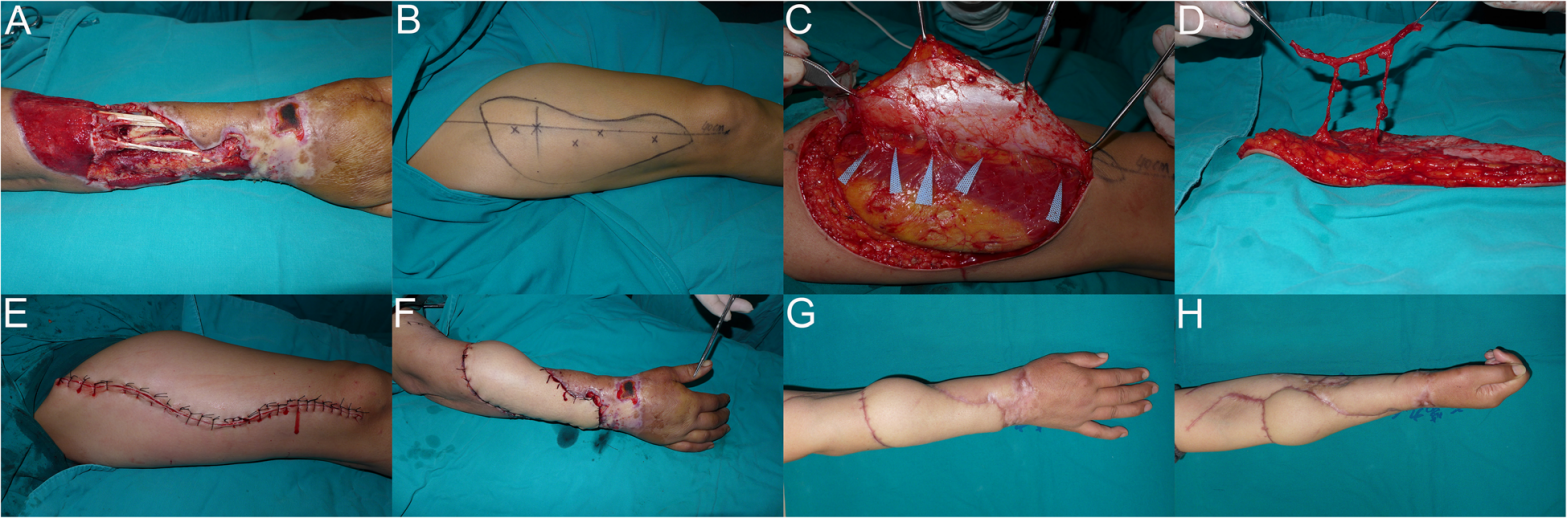
Case 1, a 48-year-old woman. **a** The defect on the right was on the forearm with the size 7 × 28 cm^2^. **b**–**d** According to the guide points (black cross) marked on the patient’s right thigh, an appropriate flow-through flap (size 7 × 28 cm^2^) was designed. **e** Primary closure of the donor site. **f** Flap covered the wound after anastomoses were complete. **g**–**h** Appearance at 15-month follow-up

A 48-year-old woman suffered a rolling-machine crush injury of her right forearm, with extensor side and palm skin avulsion, radius fracture, and devascularization of the hand and forearm. Emergency exploration revealed segmental ulnar artery defects; however, the nerve was spared. The extensor group muscles were crushed and partially disrupted. Bone reduction and internal fixation using plates were performed. A free right anterolateral thigh flap using the flow-through technique with a segment of the descending branch of the lateral circumflex femoral artery about 12 cm long was harvested 3 weeks later. The anterolateral thigh flap was about 7 × 28 cm^2^ in size. An iliac bone graft was performed for the radius defect 7 months postoperatively. Range of motion of the right wrist was 30° in flexion, with wrist extension of about 40° at 15 months postoperatively (Fig. 1).

### Case 2

**Fig. 2:**
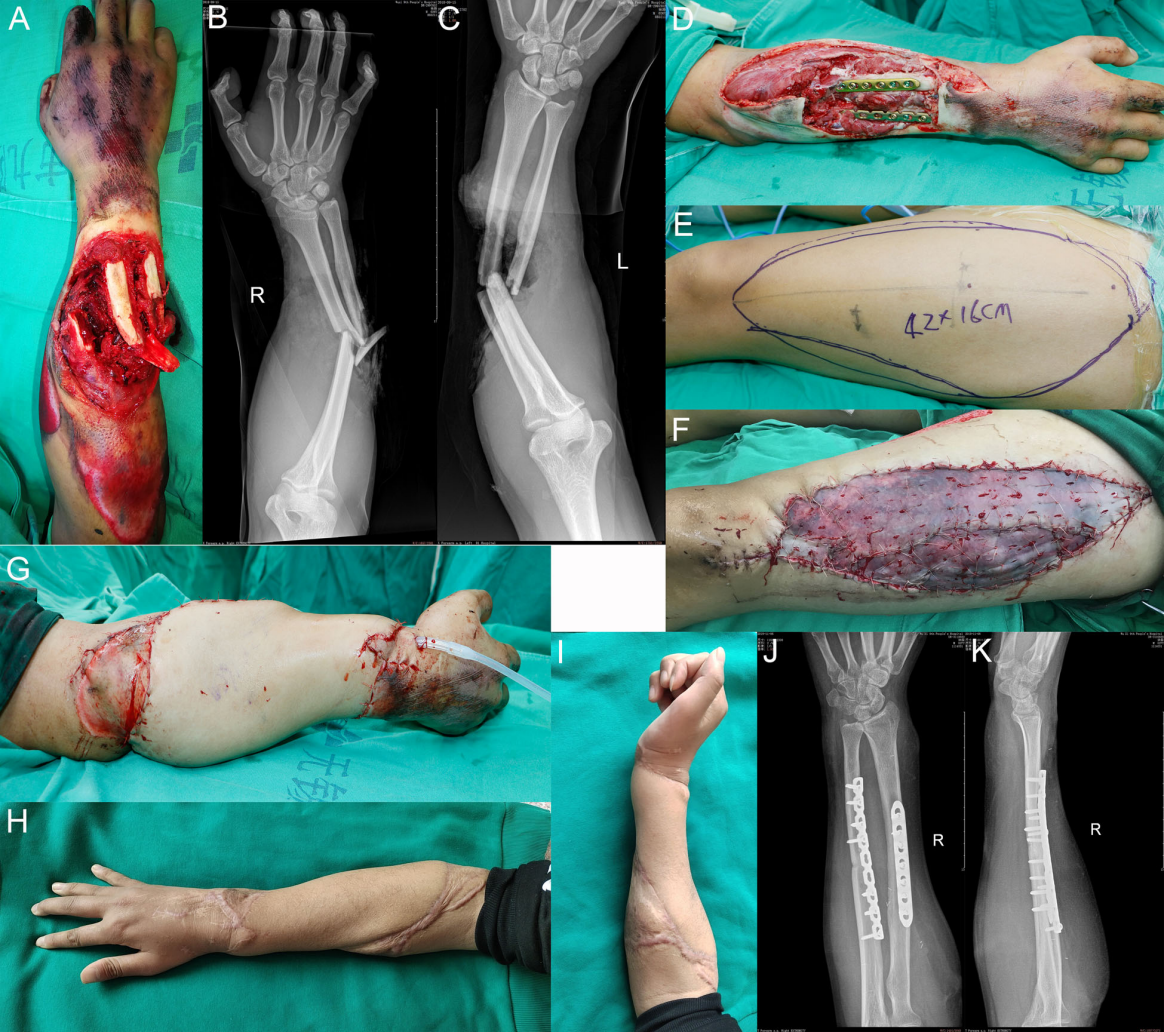
Case 2, a 23-year-old man. **a** Right arm crush injury with soft tissue defect with cortical defect in the dorsal part of ulna and radius. **b**, **c** Both ulnar and radial fractures. **d** The defect on the right was on the forearm with the size 16 × 42 cm^2^. **e**–**g** Flap covering the reconstructed soft tissue. **h**–**i** Appearance at 18-month follow-up. **j**–**k** The X-ray showed fracture healing at 15 months after surgery.

A 23-year-old man suffered a crush injury of his right forearm, with extensor skin avulsion, cortical defect in the dorsal part of ulna and radius, and severe periosteum exfoliation. There is a severe damage to the extensor tendons and muscles. Emergency debridement and exploration were arranged. All devascularized tissues were debrided as completely as possible. Fractures were internally fixed using dynamic compression plates. A free left anterolateral thigh flap about 42 cm long was harvested 5 days later. The anterolateral thigh flap was about 16 × 42 cm^2^ in size. The donor site was grafted with split-thickness skin. The flexor tendon transposition was performed 13 months after surgery to reconstruct the function of the wrist and finger extension. The flap was soft in texture and satisfactory in appearance at 18 months after the operation (Fig. 2).

## Discussion

High-energy injuries are associated with loss of the muscle, nerve, blood vessels, and bone, making it difficult to treat the wounds after primary debridement. With the development of surgical techniques, especially microsurgery, many limbs have been preserved instead of being amputated. Therefore, the focus has shifted to how to improve the appearance and function of the preserved limbs.

From the viewpoint of functional outcomes, we partially agree with the absolute and relative indications of subacute free transfer recommended by Hwang et al. [8]. As early as 1986, those involving early covering of the wound was by Godina [4]. It was suggested that if flap transplantation should be carried out within 72 h to close the wound, the necrosis rate of the flap would be 0.75%, greatly reducing the postoperative infection rate and hospital stays. However, since 1990, several studies have shown that secondary tissue transplantation can achieve better effects. In particular, there has been application of the negative pressure wound therapy technique [9]. The benefits of negative pressure wound therapy include increased perfusion, promotion of fresh granulation tissue, promotion of angiogenesis, wound area reduction, cellular proliferation, decreased bacterial contamination, and reduction of the inhibitory effects of wound exudates [10, 11]. According to Derderian, covering the wound using tissue transplantation on the 6th and 21st day after injury has obvious advantages over other times [7]; flap necrosis rates, postoperative infection rates, incidences of osteomyelitis, and nonunion were lower and the one-stage operation time was shortened, making patients more tolerant to the secondary operations. Delayed closure of the wound prevents proliferation of anaerobic bacteria while allowing sufficient time to identify the tissue that survives during primary debridement, avoiding more radical scavenging of surviving tissue, or leaving possible necrotic tissue resulting in late wound deferred. However, the later the wound is closed, the worse the function of the affected limb, resulting in heavier psychological and economic burdens. The reasons for this can be summarized as follows: (1) Wound infections correlate with longer exposure times. Despite the fact that the VSD negative pressure sealing technique keeps the wound closed, it can only guarantee effective sealing for 3 days. (2) Long-term inflammatory stimulation causes edema and adhesion of surrounding tissues, leading to stiffness and tissue organization. (3) Long-term exposure of bony tissue often leads to nonunion, necrosis, and bone infections, increasing the difficulty of bone tissue repair at the later stages. (4) Long-term bed-rest leads to disuse atrophy of muscle and late limb dysfunction. (5) Inflammation stimulates peripheral blood vessels and nerves, causing edema and adhesions with surrounding tissue, resulting in increased graft necrosis rates of the late flap.

For severely complex upper limb wounds, selecting the tissue with adequate blood supply to cover the wound is most important [12, 13]. In our study, three kinds of flap were used to reconstruct upper limbs. The lateral arm flap could only be transplanted to repair smaller defects in the forearm or hand because of tissue limitations. The latissimus dorsi musculocutaneous flap provides sufficient tissue to repair the extensive soft tissue defects; however, patients later complained not only of postoperative bloated appearance, but also of the heavier limb with limited flexibility of the affected limbs. The anterolateral thigh flap also called a “universal flap,” designed to include several tissue components, has been widely used to repair soft tissue defects [14]. We used the anterolateral thigh flap without fascia lata to repair the wounds without obvious bone exposure and infection, reducing damage to the donor site, while simultaneously improving the appearance of both donor and recipient site. For the patients with wide defect areas, lobulated flaps can be designed to change the width to length, ensuring one-stage closure of the donor area. This avoids several complications, including scarring, adhesions, and contour defects caused by skin grafting. If the damage involves the trunk vessels in the recipient area, flow-through flaps are the best choice because they provide simultaneous arterial reconstruction and soft tissue coverage [15]. The latissimus dorsi flap is most suitable for patients who need restoration of elbow function, because this flap provides good dynamic muscles for the flexor and extensor muscle group [11].

Because of inter-individual variations in perforator anatomy, preoperative vascular mapping was introduced to help identify the dominant perforator. In our study, we performed computed tomography angiography (CTA) or color Doppler sonography (CDS) to map perforators before applying an ALT perforator flap to repair soft tissue defects. According to our previous study [16], CTA had a higher sensitivity than CDS for detecting vessels. Therefore, we typically used CTA first to create clear 3D images of the vessels and surrounding structures, as well as to mark the skin. If the patient was allergic to iodine or had renal insufficiency, they would undergo CDS to determine the position, path, caliber, and quality of the perforator vessels, and to mark the skin. We depended on the results of CTA and CDS to identify the best perforators to design the ALT perforator flaps. If the perforators are located precisely, the surgeon can identify the path of the perforator in the surrounding area and minimize incisions of the muscle and deep fascia during the operation, thereby reducing complications at the donor site. This also makes the surgeon’s task easier.

Of the 35 patients we treated, individualized treatments were developed according to the patient’s condition and injury, as well as their condition on admission. In the subacute stage, the wound was closed by tissue transplantation. Therefore, we believe that subacute wound closure, if possible within 5–30 days after emergency surgery, remains a better choice. Emergency flap transplantation is not recommended unless there are important vessels and nerves that must be repaired. Mundi suggested that primary wound closure is best for fractures with less severe soft tissue injuries, to allow for tension-free closure. For those injuries requiring delayed closure, definitive coverage should not be delayed beyond 7 days, even in the setting of negative pressure wound therapy [17]. We believe that, for patients with severe open fracture of upper limb, phased treatment not only effectively reduces the infection rate, but also allows more choices of skin flap in the subacute stage. In our series, no patient underwent amputation. The two lost partial flaps were an anterolateral thigh flap and a latissimus dorsi flap, both because of deficient blood supply (5.71%). Traumatized wound infection occurred in three patients (8.57%). Two of them resulted from progressive muscle and soft tissue necrosis of the wound; however, the patient with superficial infection healed after five change dressings at the bedside. Zoran et al. reported that the main reason for infection was most often nosocomial infection [18], in agreement with our series.

Patients with severe open fractures of the upper extremity often suffer severe trauma, requiring frequent operations and long periods of treatment. If there is difficulty in performing free flap transplantation, flap necrosis may lead to amputation. Therefore, it is very important to evaluate and select blood vessels in the recipient area before surgery. First, we should evaluate the patient’s condition comprehensively according to the location of the wound and the degree of injury. Prior to surgery, CTA or B-ultrasound should be performed to verify vascular patency and diameter. During surgery, the quality of blood vessels should be accurately evaluated to avoid scarring vessels. Furthermore, heparin should be used to flush the vessels repeatedly during the operation to judge its quality and patency.

## Conclusions

Our findings support the notion that devastating upper wounds can be successfully reconstructed in the subacute period with low flap failure, infection, and amputation rates. Treatment of upper open fractures should focus on the methodology of soft tissue coverage rather than the operative timing for early coverage. For extensive soft tissue defects of the upper limb without obvious bone exposure, using an anterolateral thigh flap satisfies both appearance and function with negligible complications. For restoration of elbow function, the latissimus dorsi flap is preferable because it provides good dynamic muscle for the flexor and extensor muscle group. Preoperative perforator localization technology allows surgeons to accurately understand the vascular situation, thereby reducing the surgical failure rate.

## Data Availability

The datasets used and analyzed during the current study are available from the corresponding author on reasonable request.

## Abbreviations

ALT: Anterolateral thigh
CDS: Color Doppler sonography
CTA: Computed tomography angiography
VSD: Vacuum sealing drainage
